# Effectiveness of Sclerotherapy to Cure Lower Limb Chronic Venous Insufficiency Grades 1-6: A Systematic Review and Meta-Analysis

**DOI:** 10.7759/cureus.49770

**Published:** 2023-12-01

**Authors:** Dian Andriani Ratna Dewi, Abraham Arimuko, Lilik Norawati, Ratna K Dewi, Anak Agung Gde P Wiraguna, Arohid Allatib, Nabila Arkania, Farrasila Nadhira, Ni M Wiliantari

**Affiliations:** 1 Department of Dermatovenereology, Indonesian Defense University, Bogor, IDN; 2 Department of Dermatovenereology, Gatot Soebroto Army Central Hospital, Central Jakarta, IDN; 3 Department of Dermatovenereology, RKD's Principal Clinic, East Jakarta, IDN; 4 Department of Dermatovenereology, Medical Faculty, Udayana University, Bali, IDN; 5 Department of Dermatovenereology, Faculty of Military Medicine, Indonesian Defense University, Bogor, IDN; 6 Department of Dermatovenereology, Faculty of Medicine, Public Health, and Nursing, Gadjah Mada University, Yogyakarta, IDN; 7 Department of Dermatovenereology, Ratna Dewi Principal Clinic, Bekasi, IDN

**Keywords:** randomized controlled trial, clinical trial, sclerosing agent, lower limb, systematic review, meta-analysis, effectiveness, sclerotherapy, chronic venous insufficiency

## Abstract

Chronic venous insufficiency is a medical condition that impacts the venous system in the lower limbs. The primary characteristic of this condition is the continual elevation of pressure within the leg veins due to walking, which leads to a range of associated issues, such as discomfort, swelling, alterations in the skin, and the development of ulcers. Sclerotherapy has emerged as a potentially effective treatment for chronic venous insufficiency, involving the injection of a sclerosing agent into the affected veins. This study aimed to evaluate the effectiveness of sclerotherapy in treating chronic venous insufficiency grades 1-6 in the lower limb through a systematic review and meta-analysis. The method for conducting this research is by searching articles with relevant keywords carried out through electronic databases, such as Cochrane Library, Pubmed, and Google Scholar. The researchers conducted a comprehensive search and included randomized controlled trials and clinical trials that assessed outcomes, such as patient satisfaction, clinical resolution, quality of life (QoL), and closure rates. Statistical methods were used to analyze the data. The results of the meta-analysis from 9.670 total samples showed that sclerotherapy is the most effective therapy for reducing clinical severity in lower limb venous insufficiency patients (pooled MD = -0.85, 95% CI (-1.41, -0.29), p < 0.00001, I^2^ = 99%). Sclerotherapy is the most effective therapy for increasing QoL if we use Venous Insufficiency Epidemiology and Economic Study-Quality of Life (VEINES-QOL) as a tool for measuring the QoL (VEINES-QOL scale types; pooled MD = 10.34, 95% CI (8.78, 11.90), p < 0.00001, I^2^ = 0%). Additionally, sclerotherapy is a more effective therapy for increasing QoL compared to placebo (pooled MD = -1.64, 95% CI (-2.60, -0.67), p = 0.002, I^2^ = 90%), deferred ablation (pooled MD = -0.22, 95% CI (-0.40, -0.03), p = 0.02), and ligation therapy (pooled MD = -1.29, 95% CI (-1.62, -0.97), p = 0.00001). It is also the most effective therapy for increasing the closure rate for 12-month duration outcome measures (pooled RR = 0.72, 95% CI (0.55, 0.94), p = 0.001, I^2 ^= 91%). However, high heterogeneity was observed in the meta-analysis results, indicating the need for further research to address this variability. This study contributes to the existing body of knowledge on the treatment options for chronic venous insufficiency and highlights the potential of sclerotherapy as an effective treatment modality.

## Introduction and background

Chronic venous insufficiency (CVI) is a prevalent vascular disorder that affects a significant proportion of the global population. It is characterized by impaired venous return in the lower limb, resulting in symptoms such as leg pain, swelling, varicose veins, and changes in the skin. The prevalence of CVI varies among different populations, with estimates suggesting that it affects approximately 20-30% of adults in the general population. However, specific subgroups, such as older individuals and those with a family history of venous disease, may have a higher prevalence [1].

CVI has a substantial impact on patients' quality of life (QoL), causing physical discomfort, functional limitations, and psychological distress [2,3]. Additionally, it significantly burdens healthcare systems due to associated healthcare costs and productivity losses [4]. Therefore, effective management strategies for CVI are crucial to alleviate symptoms, improve patients' QoL, and reduce the economic burden.

Various treatment options have been explored for CVI, including conservative measures such as compression therapy, lifestyle modifications, and pharmacological interventions [5,6]. However, these approaches may only sometimes yield satisfactory outcomes, particularly in advanced cases of CVI. In such instances, more invasive interventions such as surgery and endovenous ablation techniques have traditionally been employed [4].

Sclerotherapy has emerged as a minimally invasive treatment modality for CVI [4]. It involves injecting a sclerosing agent into the affected veins, causing them to collapse and eventually be absorbed by the body [7]. Sclerotherapy can be performed using different techniques, including liquid sclerotherapy, foam sclerotherapy, and ultrasound-guided sclerotherapy. This procedure has gained popularity due to its effectiveness, safety, and cost-effectiveness compared to traditional surgical interventions [8].

While the efficacy of sclerotherapy in treating CVI has been demonstrated in several studies, there is still a need for a comprehensive evaluation of its effectiveness [9]. Previous systematic reviews and meta-analyses have provided valuable insights into the outcomes of sclerotherapy in CVI. However, these studies have focused on specific patient populations or compared sclerotherapy with other interventions, limiting the generalizability of their findings [10].

Therefore, this study aims to conduct a systematic review and meta-analysis to evaluate the overall effectiveness of sclerotherapy in treating CVI grades 1-6 in the lower limb. By synthesizing the available evidence, this study aims to provide a comprehensive evaluation of the effectiveness of sclerotherapy.

Methods

Research Methodology

We conducted this systematic review and meta-analysis study using PRISMA (Preferred Reporting Items for Systematic Reviews and Meta-Analyses) guidelines and the Cochrane Handbook for Systematic Reviews of Interventions, version 6.3.2022. We searched for literature published from 2000 to 2023.

Eligibility Criteria

Before conducting the literature search, inclusion and exclusion criteria were established to determine the relevance of the data. The inclusion criteria are 1) randomized studies, including randomized controlled trials and clinical trials; 2) patients with lower limb chronic venous insufficiency grades C1-C6 by the comprehensive classification system (CEAP); 3) with and without sclerotherapy interventions; 4) assessing outcomes patient satisfaction, clinical resolution, QoL, side effects, clinical improvement, pain, skin hyperpigmentation, and 5) using the English language.

Exclusion criteria were 1) the study being not a clinical trial; 2) combining sclerotherapy with other therapies in the same population; 3) the patient with another comorbid and not lower limb venous insufficiency; and 4) using incompatible language. The title and abstracts of the included paper were selected by three independent reviewers (AA, AP, and LN), while the other authors were consulted (DD, NW) to resolve disagreements.

Standard of References

A randomized controlled trial demonstrating the sclerotherapy effect on lower limb venous insufficiency was used as a reference (Table 1).

**Table 1:** Clinical question. This table was created by AA, one of the authors of this article.

Patient	Intervention	Comparison	Outcomes
Venous insufficiency patient with grade C1-C6 CEAP Classification in Lower Limb	Sclerotherapy (Saline-sclerotherapy, Foam-sclerotherapy, Glycerin-Sclerotherapy, Ultrasound-Guided Sclerotherapy)	Placebo, Standard Therapy	Patient Satisfaction, Clinical Resolution, Quality of Life, Adverse Event, Clinical Improvement, Pain, Hyperpigmentation of Skin

Search Strategy

**Figure 1:**
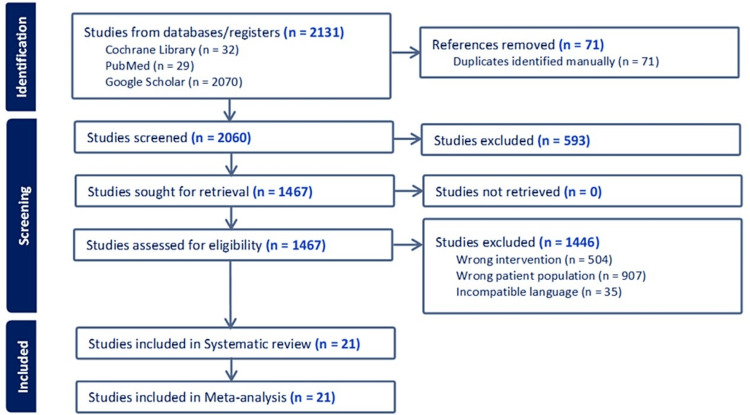
Preferred reporting items for systematic reviews and meta-analyses (PRISMA) flow diagram. This figure was created by LN, one of the authors of this article.

As shown in Figure 1, the literature research was conducted using the following databases: Cochrane, PubMed, and Google Scholar. The searches were conducted from 21 October to 1 November 2023. All terms were according to the MeSH (Medical Subject Headings) browser. Keywords were used in the search field with the Boolean operator. Specific keywords are used by following the guidelines of boolean operator keywords, namely, (((Telangiectasia OR reticular vein OR ulcer OR varicose vein OR spider vein (MeSH Terms)) AND (Sclerotherapy OR saline-sclerotherapy OR foam-sclerotherapy OR glycerin-Sclerotherapy OR ultrasound-Guided Sclerotherapy(MeSH Terms))) AND (patient satisfaction OR clinical resolution OR quality of life OR adverse event OR side effect OR clinical improvement OR pain OR hyperpigmentation of skin)) AND (lower limb OR great saphenous vein OR superficial OR small saphenous vein OR perforator vein).

Data Extraction and Analysis

The data extracted from the selected studies were author, year, country, sample size, type of control, type of trial, grade of venous insufficiency, duration of measure outcome, changes in QoL, clinical severity outcomes, and closure rate. Meta-analysis was performed using RevMan version 5.4 (Cochrane Collaboration, 2020, Copenhagen, Denmark).

Risk of Bias in Individual Studies (Qualitative Synthesis)

**Figure 2:**
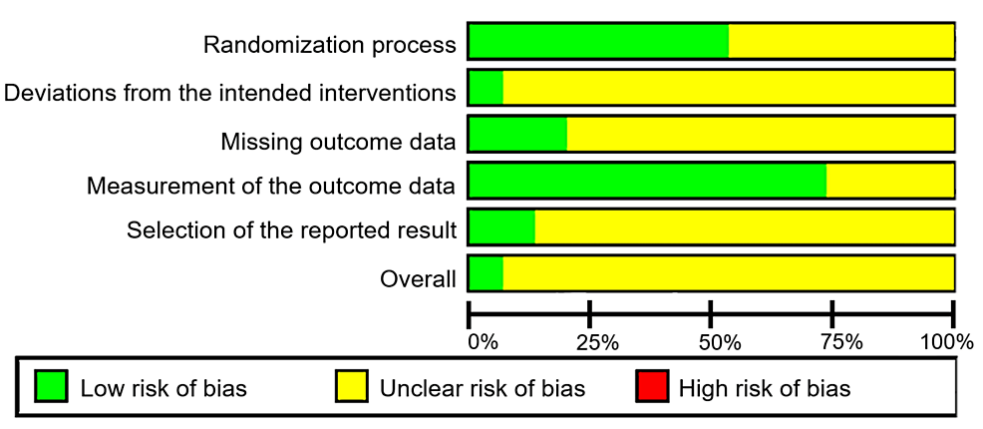
Randomized controlled trial risk of bias. This figure was created by NMW, one of the authors of this article.

**Figure 3:**
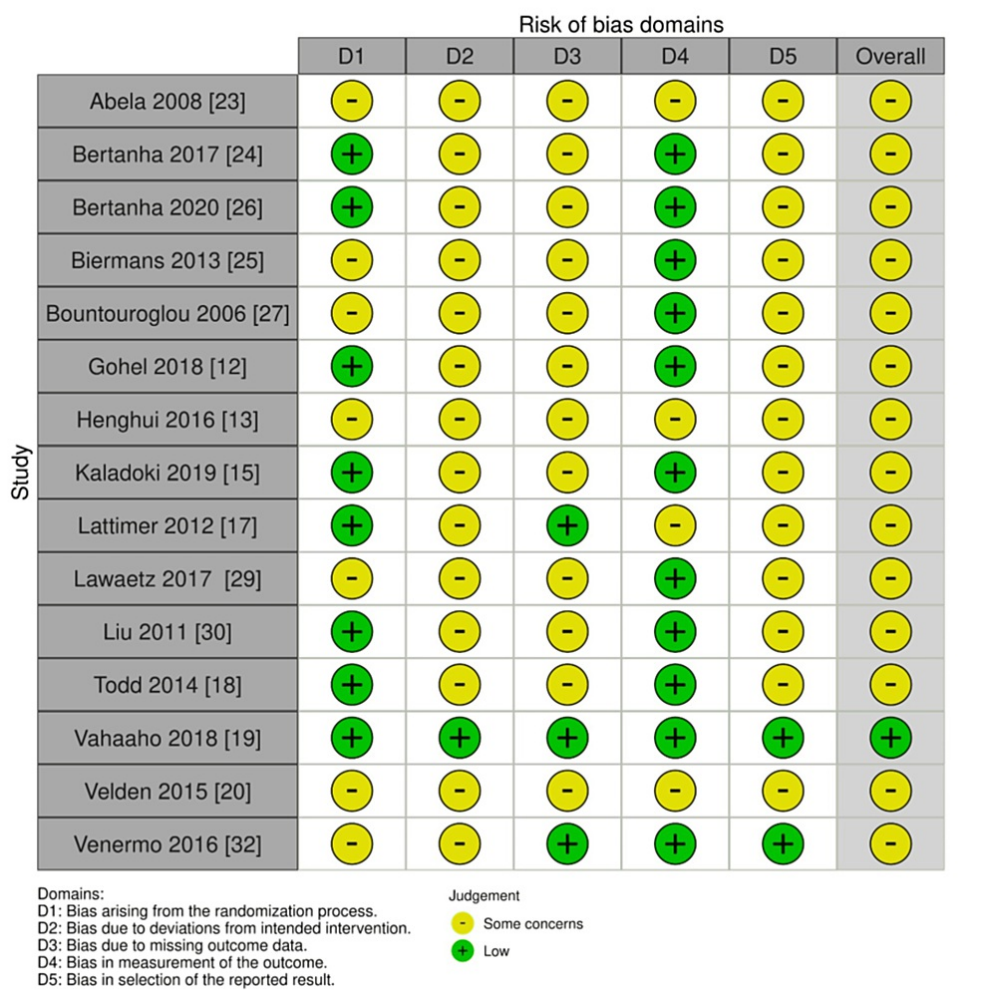
Randomized controlled trial risk of bias. This figure was created by WG, one of the authors of this article.

**Figure 4:**
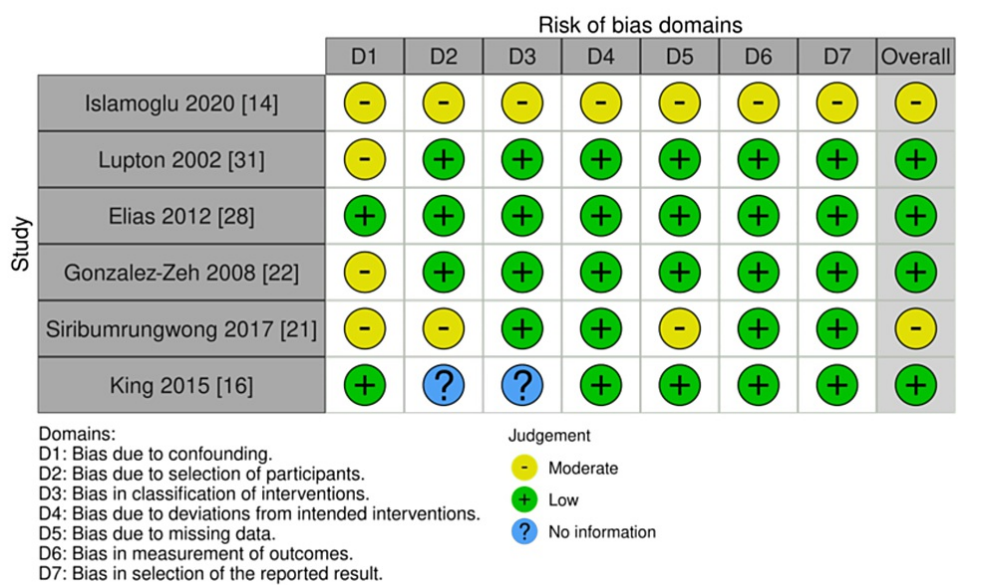
Clinical trial risk of bias. This figure was created by WG, one of the authors of this article.

The quality of the selected randomized controlled trials was evaluated using the Risk of Bias Tool for Randomized Trials version 2 (RoB2), which consists of five domains and 28 signaling questions related to the randomized process, intervention, outcome data, and reported results. On the other hand, the quality of the selected clinical trials was assessed using the Risk Of Bias In Non-randomized Studies of Interventions (ROBINS-I), which consists of seven domains, including confounding, selection of participants, classification of intervention, deviations from intended interventions, missing data, measurement of outcomes, and selection of reported results. The quality assessment was conducted by three independent reviewers (AA, DD, NW), and the results were recorded using the domain file bias (.xlsx). Using a traffic light system, the findings were summarized and displayed on the ROBVIS website. The score was presented based on algorithms for proposed judgment RoB2, such as low risk, some concerns, and high risk, and for ROBINS-I, judgments are low, moderate, serious, critical, and no information. An indication of study quality is shown in Figures 2-3. A summary of the studies included in this review can be seen in Figure 4.

Quantitative Data Synthesis (Meta-Analysis)

Review Manager 5.4.1. (Nordic Cochrane Center, Cochrane Collaboration, Copenhagen, Denmark)) was used for quantitative analysis of the data obtained. For the QoL and clinical severity outcomes, we used mean difference (MD) and standard deviation (SD) for the intervention and control group before and after treatment, which were extracted from previously included studies. The data used were of continuous type. The statistical method used was inverse variance. For the closure rate outcomes, we used relative risk (RR) for the intervention and control group after treatment were extracted from previously included studies. The data used were of dichotomous type. The statistical method used was Mantel-Haenszel. Furthermore, the model analysis used was fixed effect model (FEM) if the level of heterogeneity (I^2^) < 50% and with random effect model (REM) if the level of heterogeneity (I^2^) ≥ 50%. MD and SD for change from baseline using sclerotherapy for venous insufficiency patients were assessed as the primary outcomes guiding the statistical analysis, as indicated by significant effects on QoL and clinical severity outcomes. Standardized MD (SMD) with study and total confidence intervals of 95% (CI) were used to quantify the effects.

## Review

Result

From all inclusion journals published from 2000 to 2023, there are 9.670 total samples divided into an intervention group of 5.450 and a control group of 4.220 patients/leg. We extracted 21 studies conducted in different countries. Five studies were conducted in the UK and four studies in the USA, each of them has two studies carried out in Finland, Netherlands, Brazil, and China, and each conducted one research in Turkey, Denmark, Chile, and Thailand.

Sclerotherapy is a commonly used treatment for varicose and telangiectatic leg veins, involving injecting a sclerosing agent into the affected veins to induce fibrosis and subsequent closure. This minimally invasive procedure has been shown to effectively improve the appearance of veins and reduce symptoms associated with venous insufficiency [8]. However, there is ongoing debate regarding the optimal choice of sclerosing agent and the need for additional interventions alongside sclerotherapy. Several studies have compared the outcomes of interventions with and without sclerotherapy, aiming to determine the efficacy and safety of this treatment modality [11]. In this review, we examined the results of these studies and explored the benefits and limitations of sclerotherapy compared to other interventions.

In our review, we divided the meta-analyses into subgroups to provide more specific results depending on the data available in the inclusion journals. The results of our meta-analysis were categorized by outcome type, scale or instrument used, and treatment period.

The first subgroup was divided by outcome type: QoL, clinical severity, and closure rate. Nine inclusion journals provided results on the effect of sclerotherapy on the QoL in venous insufficiency patients [12-20]. As for the assessment of clinical severity outcomes, we used 10 available inclusion journals [12-14,16-18,20-22]. As for the evaluation of closure rate outcomes, we used 17 available inclusion journals [12,16-20,22-32].

Sclerotherapy is a minimally invasive treatment for varicose and telangiectatic leg veins that injects a sclerosing agent into the affected veins. The sclerosing agent causes irritation and inflammation in the vein walls, leading to their closure and eventual absorption by the body. This process is known as fibrosis and results in the elimination of the treated veins [33]. Based on the articles we reviewed, we found 21 articles that examined the effectiveness of sclerotherapy compared with various other therapies in treating varicose veins. A summary of each inclusion journal can be seen in the extraction table in Table 2.

**Table 2:** Data extraction. N/A = Not administrated NI = No Information * = Pre-intervention – post-intervention, if using a questionnaire that is of good value the higher the score. Post-intervention - pre-intervention, if using a questionnaire that is of good value, the lower the score. This table was created by FN, one of the authors of this article.

No	Author, Year	Title (Country)	Study Design	Number of Participants (Trial /Control)	Type of Control	Type of Trial	Grade of Venous Insufficiency (name of vein)	Duration of Outcome Measures	Changes Quality of Life Outcomes, Mean (SD)*			Clinical Severity Outcomes, (After - Before), Mean (SD)			Closure rate, number of participants		
									Trial	Control	Tools	Trial	Control	Tools	Trial	Control	Tools
1.	Kaladoki et al., 2019 [15]	The Discord Outcome Analysis (DOA) as a Reporting Standard at Three Months and Five Years in Randomised Varicose Vein Treatment Trials (UK)	Randomized Controlled Trial	100 (50/50)	Endovenous Laser Ablation (EVLA)	Ultrasound Guided Foam Sclerotherapy (UGFS)	Grade C2 CEAP (Great saphenous vein)	60 months	-12.8 (14.0748)	-13.8 (15.4822)	Aberdeen Varicose Vein Questionnaire (AVVQ)	N/A	N/A	N/A	N/A	N/A	N/A
2.	Liu et al., 2011 [30]	Ultrasound-guided foam sclerotherapy of the great saphenous vein with sapheno-femoral ligation compared to standard stripping: a prospective clinical study (China)	Randomized Controlled Trial	60 (30/30)	Standard Stripping Surgery	Ultrasound-Guided Foam Sclerotherapy (UGFS) with Sapheno-femoral junction ligation (SFJ)	Grade C2 CEAP (Great saphenous vein)	6 months	N/A	N/A	N/A	N/A	N/A	N/A	25	27	Ultrasound imaging
3.	Todd et al., 2014 [18]	The VANISH-2 study: a randomized, blinded, multicenter study to evaluate the efficacy and safety of polidocanol endovenous microfoam 0.5% and 1.0% compared with placebo for the treatment of sapheno-femoral junction incompetence (USA)	Randomized Controlled Trial	123 (67/56)	Placebo (Agitated Diluent Solution)	Polidocanol Endovenous Microfoam (PEM)	Grade C2-C6 CEAP (Great saphenous vein)	2 months	-14.5 (6.2)	-4.2 (2.1)	Modified Venous Insufficiency Epidemiological and Economic Study-Quality of Life (VEINES-QOL)	-6.4 (2.9)	-0.9 (2.6)	Venous Clinical Severity Score (VCSS)	58	1	Duplex Ultrasound Respons
4.	Lupton et al., 2002 [31]	Clinical Comparison of Sclerotherapy Versus Long-Pulsed Nd: YAG Laser Treatment for Lower Extremity Telangiectases (USA)	Clinical Trial	20 (10/10)	Long-Pulse Nd: YAG laser treatment sclerotherapy	Sodium Tetradecyl Sulfate (STS)	Grade C1 CEAP (NI)	3 months	N/A	N/A	N/A	N/A	N/A	N/A	4	7	Clinical Improvement Scale
5.	Elias et al., 2012 [28]	Mechanochemical Tumercentless Endovenous Ablation: Final Results of the Initial Clinical Trial (USA)	Clinical Trial	29 (15/14)	Mechanochemical ablation using Clarivein	Ultrasound Guided Foam Sclerotherapy (UGFS) using ClariVein	Grade C2 CEAP (Great saphenous vein)	6 months	N/A	N/A	N/A	N/A	N/A	N/A	14	13	Ultrasound imaging
6.	Gohel et al., 2018 [12]	A Randomized Trial of Early Endovenous Ablation in Venous Ulceration (UK)	Randomized Controlled Trial	449 (223/226)	Endovenous ablation deferred	Ultrasound-guided foam sclerotherapy (UGFS)	Grade C6 CEAP (NI)	1.5 months	-2.1 (4.4)	-1.2 (3.9)	Aberdeen Varicose Vein Questionnaire (AVVQ)	-5.3 (4.7)	-3.1 (4.4)	Venous Clinical Severity Score (VCSS)	167	113	Kaplan-Meier Methode
7.	Bertanha et al., 2017 [24]	Sclerotherapy for Reticular Veins in the Lower Limbs A Triple-Blind Randomized Clinical Trial (Brazil)	Randomized Controlled Trial	106 (51/55)	Placebo (75% hypertonic glucose alone)	Polidocanol diluted in 70% Hypertonic Glucose (HG)	Grade C1 CEAP (reticular vein)	2 months	N/A	N/A	N/A	N/A	N/A	N/A	24	14	Linear measurements using ImageJ software
8.	Biemans et al., 2013 [25]	Comparing endovenous laser ablation, foam sclerotherapy, and conventional surgery for great saphenous varicose veins (Netherlands)	Randomized Controlled Trial	155 (77/78)	Endovenous Laser Ablation (EVLA)	Ultrasound Guided Foam Sclerotherapy (UGFS)	Grade C2-C5 CEAP (Great saphenous vein)	12 months	NI	NI	Chronic Venous Insufficiency Quality-of-Life Questionnaire (CIVIQ)	N/A	N/A	N/A	56	69	Ultrasound imaging
9.	Gonzalez et al., 2008 [22]	Endovenous laser and echo-guided foam ablation in great saphenous vein reflux: one-year follow-up results (Chile)	Clinical Trial	98 (53/45)	Endovenous laser ablation (EVLA)	Echo-guided chemical ablation with foam (ECHA)	Grade C2-C6 CEAP (Great saphenous vein)	12 months	N/A	N/A	N/A	-0.0305 (0.491)	-1.1712 (1.0987)	Venous Clinical Severity Score (VCSS)	41	42	duplex ultrasound scan
10.	Lawaetz et al., 2017 [29]	Comparison of endovenous ablation techniques, foam sclerotherapy, and surgical stripping for great saphenous varicose veins. An extended 5-year follow-up of an RCT (Denmark)	Randomized Controlled Trial	288 (144/144)	Endovenous laser ablation (EVLA)	Ultrasound-guided foam sclerotherapy (UGFS)	Grade C2-C6 CEAP (Great saphenous vein)	60 months	NI	NI	Aberdeen Varicose Vein Symptom Severity Score (aVVSS)	N/A	N/A	N/A	107	99	Ultrasound Imaging
11.	Bertanha et al., 2020 [26]	Polidocanol Plus Glucose Versus Glucose Alone for the Treatment of Telangiectasias: Triple Blind, Randomised Controlled Trial (PG3T) (Brazil)	Randomized Controlled Trial	98 (51/47)	Placebo (75% hypertonic glucose alone)	Polidocanol diluted in 70% Hypertonic Glucose (HG)	Grade C1 CEAP (NI)	2 months	N/A	N/A	N/A	N/A	N/A	N/A	42	30	Ultrasound Imaging
12.	King et al., 2015 [16]	Treatment of Truncal Incompetence and Varicose Veins with a Single Administration of a New Polidocanol Endovenous Microfoam Preparation Improves Symptoms and Appearance (USA)	Clinical Trial	105 (50/55)	Placebo (Agitated diluent solution)	Polidocanol endovenous microfoam (PEM) 1%	Grade C2 CEAP (sapheno-femoral junction)	2 months	-19.25 (31)	-7.67 (13.53)	VEINES-QOL/Sym (Varicose Veins Quality of Life/Symptoms) instrument-3.7	7 (6.1)	-0.75 (1.32)	Revised Venous Clinical Severity Score (rVCSS)	24 (n of trial is 57)	3 (n of control is 56)	Duplex Ultrasound
13.	Lattimer et al., 2012 [17]	Cost and Effectiveness of Laser with Phlebectomies Compared with Foam Sclerotherapy in Superficial Venous Insufficiency. Early Results of a Randomised Controlled Trial (UK)	Randomized Controlled Trial	100 (50/50)	Endovenous laser ablation (EVLA) with phlebectomies	Ultrasound-guided foam sclerotherapy (UGFS)	Grade C2-C6 CEAP (Great saphenous vein)	3 months	-12.6 (3.975)	-14.2 (2.425)	Aberdeen varicose vein questionnaire (AVVQ)	-5 (0.75)	-5 (0.75)	Venous Clinical Severity Score (VCSS)	34	37	Duplex ultrasound
14.	Abela et al., 2008 [23]	Reverse Foam Sclerotherapy of the Great Saphenous Vein with Sapheno-Femoral Ligation Compared to Standard and Invagination Stripping: a Prospective Clinical Series (UK)	Randomized Controlled Trial	57 (27/30)	Invagination therapy	Foam sclerotherapy	Grade C2-C3 CEAP (sapheno-femoral junction and Great saphenous vein)	0.5 months	N/A	N/A	N/A	N/A	N/A	N/A	26	21	Duplex scanning
15.	Bountouroglou et al., 2006 [27]	Ultrasound-guided Foam Sclerotherapy Combined with Sapheno-femoral Ligation Compared to Surgical Treatment of Varicose Veins: Early Results of a Randomised Controlled Trial (UK)	Randomized Controlled Trial	58 (30/28)	Sapheno-femoral ligation	Ultrasound-guided foam sclerotherapy (UGFS)	Grade C2-C6 CEAP (sapheno-femoral junction and Great saphenous vein)	3 months	NI	NI	Aberdeen Vein Questionnaire (AVVQ)	-4 (1.25)	-4 (1)	Venous Clinical Severity Score (VCSS)	26	26	Ultrasound Imaging
16.	Venermo et al., 2016 [32]	Randomized clinical trial comparing surgery, endovenous laser ablation and ultrasound-guided foam sclerotherapy for the treatment of great saphenous varicose veins (Finland)	Randomized Controlled Trial	145 (72/73)	Endovenous laser ablation (EVLA)	Ultrasound-guided foam sclerotherapy (UGFS)	Grade C2-C4 CEAP (Great saphenous vein)	12 months	NI	NI	Aberdeen Varicose Vein Severity Score (AVVSS)	N/A	N/A	N/A	37	71	Duplex scanning
17.	Siribumrungwong et al., 2017 [21]	Quality of life after great saphenous vein ablation in Thai patients with great saphenous vein reflux (Thailand)	Clinical Trial	56 (23/33)	Radiofrequency ablation (RFA)	Ultrasound-guided foam sclerotherapy (UGFS)	Grade C2-C3 CEAP (Great saphenous vein)	1 month	NI	NI	EuroQol descriptive system (EQ-5D) questionnaire	-1 (2.0)	-1 (2.0)	Venous Clinical Severity Score (VCSS)	N/A	N/A	N/A
18.	van der Velden et al., 2015 [20]	Five-year results of a randomized clinical trial of conventional surgery, endovenous laser ablation and ultrasound-guided foam sclerotherapy in patients with great saphenous varicose veins (Netherlands)	Randomized Controlled Trial	120 (62/58)	Endovenous laser ablation (EVLA)	Ultrasound-guided foam sclerotherapy (UGFS)	Grade C2 CEAP (Great saphenous vein)	60 months	-12.6 (6.1)	-12.6 (6.2)	Chronic Venous Insufficiency quality-of-life Questionnaire (CIVIQ)	-3.8 (2.1)	-4.2 (2.0)	Venous Clinical Severity Score (VCSS)	42	54	Duplex imaging
19.	Vahaaho et al., 2018 [19]	Five-year follow-up of a randomized clinical trial comparing open surgery, foam sclerotherapy, and endovenous laser ablation for great saphenous varicose veins (Finland)	Randomized Controlled Trial	116 (59/57)	Endovenous laser ablation (EVLA)	Ultrasound-guided foam sclerotherapy (UGFS)	Grade 2-6 CEAP (Great saphenous vein)	60 months	-1.6 (0.92)	-0.9 (1.0)	Aberdeen Varicose Vein Severity Score (AVVSS)	N/A	N/A	N/A	30	51	Ultrasound imaging
20.	Islamoqlu, 2020 [14]	Varicose vein surgery versus foam sclerotherapy to prevent extension of venous reflux with time (Turkey)	Clinical trial	7210 (4224/2986)	Stripping method	Foam sclerotherapy	NI (Great saphenous vein and sapheno-femoral junction)	60 months	-13.8 (4.4)	-14.8 (6.8)	Aberdeen Varicose Vein Questionnaire Score (AVVQ)	-5.0 (0.9)	-5.1 (0.3)	Venous Clinical Severity Score (VCSS)	N/A	N/A	N/A
21	Yin et al., 2016 [13]	Prospective Randomized Study of Ultrasound-Guided Foam Sclerotherapy Combined with GSV High Ligation in the Treatment of Severe Lower Extremity Varicosis (China)	Randomized Controlled Trial	177 (82/95)	Great saphenous vein high ligation and stripping combined with multi-stab avulsion or TIPP	Ultrasound-guided foam sclerotherapy (UGFS)	Grade C4-C6 CEAP (Grate saphenous vein and sapheno-femoral junction)	6 months	-22 (1.57)	-17 (5.04)	Aberdeen Varicose Vein Questionnaire Score (AVVQ)	-6 (0.135)	-5 (0.28)	Venous Clinical Severity Score (VCSS)	N/A	N/A	N/A

Kaladoki et al. introduced discord outcome analysis (DOA) to report results in comparative trials on the treatment of superficial venous insufficiency. Research with a single result generally provides a very high success rate, but many differences can occur when combined with other types of treatment to achieve the same goal. DOA is a method for interrogating the reporting process in research. This method will increase transparency in comparative clinical trials [15].

Liu et al. conducted a randomized controlled trial comparing ultrasound-guided foam sclerotherapy (UGFS) of the great saphenous vein (GSV) combined with sapheno-femoral junction (SFJ) ligation to standard stripping surgery. Primary endpoints were the patient recovery period, postoperative pain, QoL, and recurrence rate, and secondary endpoints were the frequency of complications in the two arms of the trial. The result of this study was UGFS combined with sapheno-femoral ligation, which involved a shorter treatment time and less postoperative discomfort and resulted in more rapid recovery compared to conventional GSV stripping [30].

Todd et al. researched to determine the efficacy and safety of polidocanol endovenous microfoam in treating symptoms and appearance in patients with SFJ incompetence due to GSV or major accessory vein reflux. The result of the study was polidocanol endovenous microfoam provides clinically meaningful benefits in treating the symptoms and appearance of patients with varicose veins [18].

King et al. conducted a multicenter study to determine whether a single administration of ≤15 mL of pharmaceutical grade polidocanol endovenous microfoam (PEM, now approved in the United States as Varithena (polidocanol injectable foam), BTG International Ltd.) could relieve symptoms and improve the appearance of varicose veins, in a patient population with symptoms of moderate to very severe superficial venous incompetence and visible varicose veins on the GSV system. This research concludes that a single administration of PEM up to 15 mL is a safe, effective, and convenient treatment for symptoms of superficial venous incompetence and the appearance of visible varicose veins in the GSV system. PEM doses of 0.5%, 1%, and 2% appear to have acceptable risk-benefit ratios [16].

Lupton et al. studied a comparison of the clinical efficacy of leg telangiectasia treatment with sodium tetradecyl sulfate sclerotherapy to long-pulsed 1064 nm Nd:YAG laser irradiation. The result of the study was that despite recent advances in laser technology for treating lower extremity telangiectases, sclerotherapy offers superior clinical effects in most cases. Laser leg vein treatment is most beneficial in patients with telangiectatic matting, needle phobia, or sclerosant allergy [31]. Meanwhile, research conducted by Gonzales-Zeh et al. compared the effectiveness of EVLA treatment and foam sclerotherapy in treating reflux of GSV closure. It was found that, overall, EVLA achieved a higher occlusion rate than echo-guided chemical ablation with foam after one year of follow-up, especially for veins above 12 mm in diameter. On the other hand, for veins measuring less than 6.5 mm, foam sclerotherapy shows higher success. Procedure-related pain was higher in the laser group (P < 0.0082). Induration, phlebitis, and ecchymosis are the most common complications [22].

Biemans et al. conducted a study comparing the anatomical success rates, frequency of major complications, and QoL improvement of EVLA, UGFS, and conventional surgery (CS) after one-year follow-up. In terms of results after one year, the anatomical success rate was the highest after EVLA (88.5%), followed by CS (88.2%) and UGFS (72.2%) (P < 0.001). After a follow-up period of one year, EVLA was as effective as CS and superior to UGFS. QoL improved significantly after treatment in all groups [25].

Lawaetz et al. compared five-year outcomes after treatment of varicose veins with endovenous radiofrequency ablation (RFA), EVLA, UGFS, or high ligation and stripping (HL/S) by assessing technical efficacy, clinical recurrence, and reoperation rates. The results obtained at five years were differences in the rates of GSV recanalization, recurrence, and reoperation between groups. More GSV recanalizations occurred after UGFS, and no differences in technical efficacy were found between other modalities over five years of follow-up. The higher frequency of clinical recurrence after EVLA and HL/S is unexplained and requires confirmation in other studies [29].

Lattimer et al. conducted a study aimed at quantifying EVLA with concurrent phlebotomy and UGFS in terms of cost and effectiveness at three weeks and three months. The assessments carried out were with the Aberdeen varicose vein questionnaire (AVVQ), venous clinical severity score (VCSS), venous filling index (VFI), visual analog seven-day pain score, and analgesia requirements. This study concluded that UGFS was 3.15 times cheaper than EVLA with comparable effectiveness but 56% (versus 6%) but required additional foam in case of recurrence [17].

Venermo et al. conducted a study to compare the effects of surgery, EVLA (with phlebotomy), and UGFS on QoL and rate of GSV occlusion 12 months after surgery. Compared with open surgery and EVLA, UGFS resulted in equivalent QoL improvement but significantly higher residual GSV reflux at 12-month follow-up [32].

Elias et al. conducted a study to assess the safety and efficacy of the ClariVein® system that uses mechanochemical ablation of the GSV. A new mechanochemical device, the catheter (ClariVein®, Madison, CT), was developed to minimize the negative aspects of endothermal ablation and ultrasound-guided sclerotherapy. Because this system does not use heat energy, the potential for nerve damage is minimized. Negative aspects eliminated with the hybrid procedure are the need for tumor anesthesia for endothermal ablation and the lower efficacy rate of ultrasound. The results are encouraging because this method eliminates the need for tumescent anesthesia. Most incompetent GCVs can be treated with this technique [28].

Abela et al. compared reverse foam sclerotherapy of GSV combined with SFJ ligation with standard stripping (Babcock) and invagination stripping (Pin) in a prospective clinical series. The results were that SFJ ligation plus reverse foam sclerotherapy of GSV was associated with less blood loss, bruising, and postoperative discomfort than either stripping techniques (p <0.001, Mann-Whitney). Standard GSV stripping and invagination stripping are not associated with major discomfort and problems in the early postoperative period. SFJ ligation and GSV reverse foam sclerotherapy result in better patient satisfaction, less bruising, postoperative discomfort, and reduced analgesic requirements [23].

Bountouroglou et al. performed a study comparing sapheno-femoral ligation, great saphenous stripping, and multiple avulsions with sapheno-femoral ligation and UGFS of the saphenous vein. What is studied is the patient's recovery period and QoL, as well as the frequency of complications. This study concluded that UGFS combined with sapheno-femoral ligation is less expensive, has a shorter treatment time, and has faster recovery than sapheno-femoral ligation, saphenous stripping, and phlebotomy [27].

Gohel et al. performed the EVRA trial to evaluate the role of early endovenous treatment of superficial venous reflux as an adjunct to compression therapy in patients with venous leg ulcers. Early endovenous ablation of superficial venous reflux resulted in faster healing of venous leg ulcers and more time free from ulcers than deferred endovenous ablation [12].

Bertanha et al. compared the efficacy and safety of two sclerosants used to treat reticular veins: polidocanol 0.2% diluted in 70% hypertonic glucose (HG) (group 1) vs 75% HG alone (group 2). The research was done because no consensus has been reached regarding the optimal sclerosant. This study showed that sclerotherapy with polidocanol 0.2% diluted in 70% HG was superior to 75% HG alone in reticular vein sclerosis, with no statistical difference for complications. Pigmentation occurred in both groups, with no statistical difference between the two. No severe side effects occurred in either group [24].

In 2020, Bertanha et al. repeated the study on a larger sample to compare the effectiveness and safety of two sclerosing agents used to treat telangiectasias of the lower extremities: 0.2% polidocanol + 70% hypertonic glucose (HG) vs. 75% HG only. Polidocanol 0.2% plus 70% HG provides better results than 75% HG alone in sclerosis telangiectasis. No serious side effects occurred, pigmentation occurred in both groups, and the duration was shorter in the group treated with polidocanol 0.2% + 70% HG [26].

Siribumrungwong et al. conducted a study to determine the QoL in Thai people after intervention for GSV reflux. QoL was measured using the EuroQol descriptive system questionnaire (EQ-5D), and patients chose to receive endovenous or surgical treatment after consultation with their surgeon. This study found that GSV ablation for GSV reflux in Thai patients with CEAP C2 and C3 categories significantly improved physical and mental QoL; patients who received endovenous procedures were found to have better initial physical quality [21].

van der Velden et al. studied patients who underwent conventional surgery, EVLA ablation, and UGFS for GSV varicose veins followed up for five years. The primary outcome was loss or absence of the treated GSV segment, and secondary outcomes were the absence of GSV reflux and changes in Chronic Venous Insufficiency Quality of Life Questionnaire (CIVIQ) and EuroQol - 5D (EQ-5D™) scores. The results were that EVLA and conventional surgery were more effective than UGFS in eliminating GSV five years after intervention [20].

Vahaaho et al. conducted research in Finland. They also evaluated the results of surgery, EVLA, and UGFS in the treatment of GSV reflux for five years. The results showed that UGFS had a much lower occlusion rate compared with open surgery or EVLA and required additional treatment [19].

Islamoqlu in Turkey compared the therapy with foam sclerotherapy and GSV stripping. It was found that foam sclerotherapy was significantly more cost-effective, more accessible to apply, and more comfortable for the patient, and there was no significant difference in clinical results between the two procedures. There is no significant difference in postoperative effectiveness between foam sclerotherapy and stripping. There is no significant difference between VCSS and AVVQ scores [14].

Yin et al. evaluated the effect of UGFS in a single session combined with GSV high ligation for severe lower extremity varicosis classified as C4-C6, compared with GSV stripping plus multistab avulsion or transilluminated powered phlebectomy (TIPP). This study concluded that UGFS combined with GSV high ligation is safe and effective for severe lower extremity varicose veins [13].

In our review, we divided the meta-analyses into subgroups to provide more specific results depending on the data available in the inclusion journals. The results of our meta-analysis were categorized by outcome type, scale or instrument used, and treatment period.

The first subgroup was divided by outcome type: QoL, clinical severity, and closure rate. Nine inclusion journals provided results on the effect of sclerotherapy on the QoL in venous insufficiency patients [12-20]. As for the assessment of clinical severity outcomes, we used 10 available inclusion journals [12-14,16-18,20-22]. As for the evaluation of closure rate outcomes, we used 17 available inclusion journals [12,16-20,22-32].

In the clinical severity subgroup, from a total of 10 inclusion studies, significant results were obtained in the intervention group (pooled MD = -0.85, 95% CI (-1.41, -0.29), p < 0,00001, I^2^ = 99%). Heterogeneity is very high, so we use random effects. This meta-analysis showed a significant association between sclerotherapy and clinical severity outcomes compared to the control.

In the QoL subgroup, from a total of nine inclusion studies, insignificant results were obtained in the control group (pooled MD = 0.10, 95% CI (-0.46, 0.66), p < 0,00001, I^2^ = 98%). Heterogeneity is very high, so we use random effects. This meta-analysis showed an insignificant association between sclerotherapy and QoL outcomes compared to the control.

Then, in the closure rate subgroup, from a total of 17 inclusion studies, insignificant results were obtained in the intervention group (pooled RR = 0.2, 95% CI (-0.17, 0.21), p < 0,00001, I^2^ = 97%). Heterogeneity is very high, so we use random effects. This meta-analysis showed an insignificant association between sclerotherapy and closure rate outcomes compared to the control.

**Figure 5:**
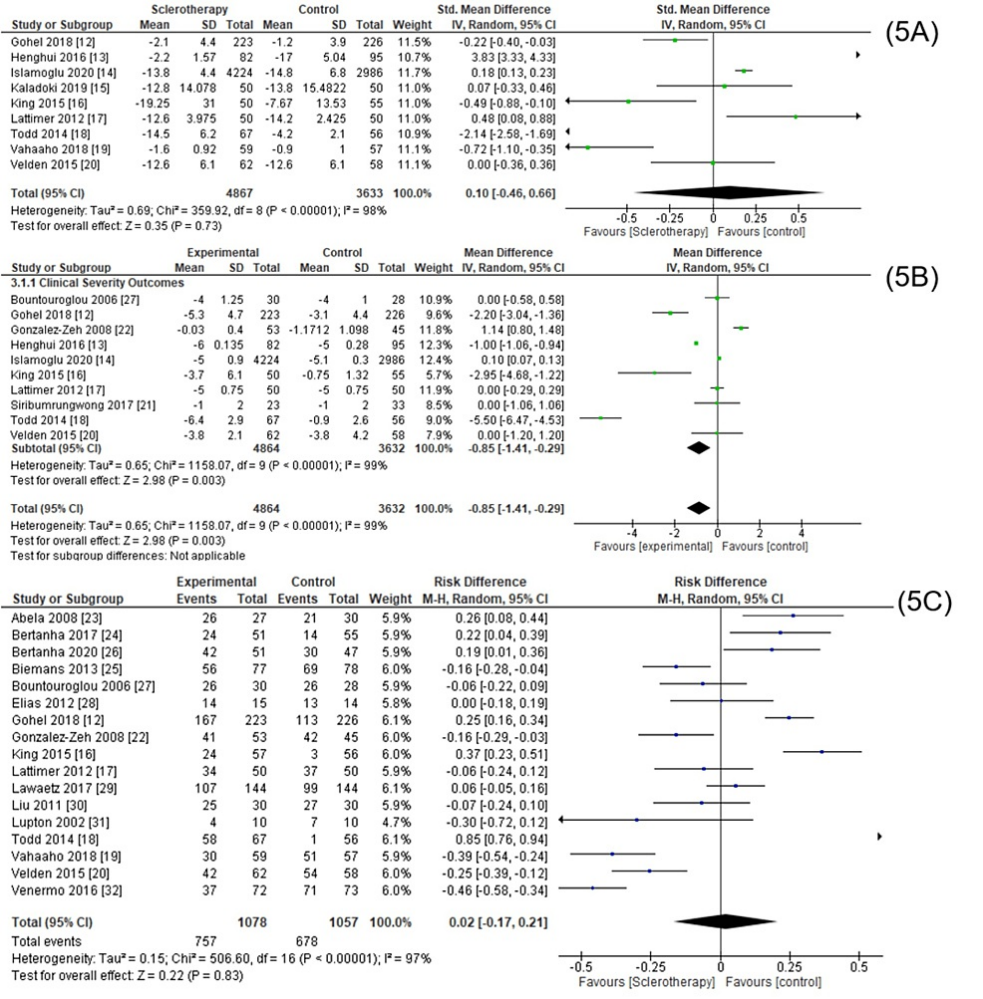
Meta-analysis based on quality of life outcomes (5A), clinical severity (5B), and closure rate (5C). This figure was created by AA, one of the authors of this article.

Unfortunately, the heterogeneity in the meta-analysis results of all inclusion journals in the QoL subgroup, clinical severity subgroup, and closure rate subgroup was very high (Figure 5). Therefore, we subdivided the subgroups into smaller subdivisions based on the instruments used for QoL subgroups, based on the type of control for QoL, clinical severity, and closure rate subgroups and based on the duration for measuring the outcome for closure rate subgroups.

We found that the data obtained from nine included studies assessed four different types of measurement scales, namely, the Aberdeen Varicose Vein Questionnaire (AVVQ), Venous Insufficiency Epidemiology and Economic Study-Quality of Life (VEINES-QOL), Chronic Venous Insufficiency Quality of Life Questionnaire (CIVIQ), and the Aberdeen Varicose Vein Severity Score (AVVS).

**Figure 6:**
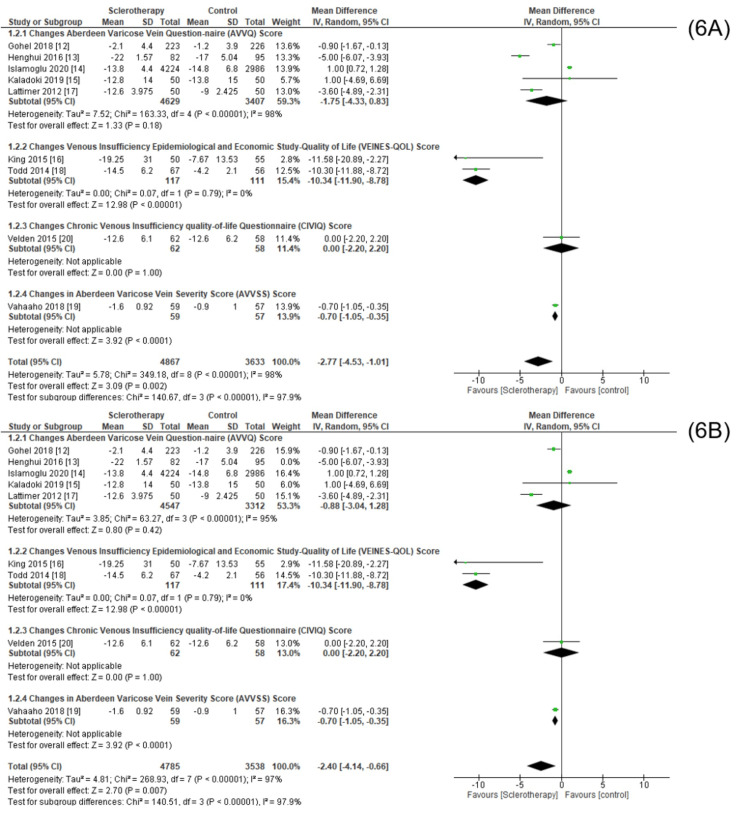
Meta-analysis based on data scale division before (6A) and after (6B) sensitivity analysis. This figure was created by RKD, one of the authors of this article.

Significant meta-analysis results in the intervention group were obtained from the VEINES-QOL scale types (pooled MD = 10.34, 95% CI (8.78, 11.90), p < 0,00001, I^2^ = 0%) (Figure 6). Since the heterogeneity in the AVVQ subdivides was still high, we tried to perform sensitivity analysis by only excluding the mean difference of one study [13]. We obtained insignificant results with high heterogeneity (Figure 6B).

Furthermore, we classified the inclusion studies from quality-of-life subgroups based on the mode of intervention or type of control. The inclusion studies were divided into five subdivisions: EVLA, stripping technique, placebo, deferred ablation, and sapheno-femoral ligation.

**Figure 7:**
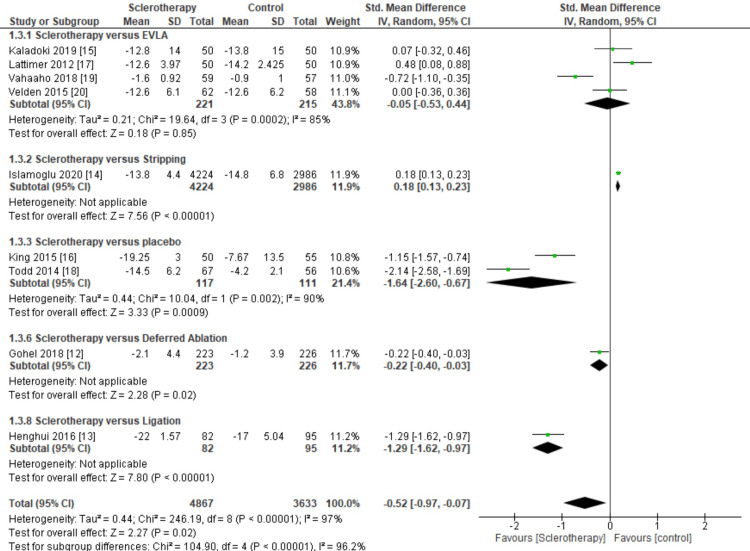
Meta-analysis based on the type of control for quality of life outcomes. This figure was created by AAr, one of the authors of this article.

From the results of the meta-analysis, the stripping provides significant results in the control group; on the other hand, placebo, deferred ablation, and ligation subgroup provide more significant developments in the intervention group, for sclerotherapy versus stripping subgroup results (pooled MD = 0.18, 95% CI (0.13, 0.23), p = 0.00001), and sclerotherapy versus placebo subgroup results (pooled MD = -1.64, 95% CI (-2.60, -0.67), p = 0.002, I^2^ = 90%), sclerotherapy versus deferred ablation subgroup results (pooled MD = -0.22, 95% CI (-0.40, -0.03), p = 0.02), and sclerotherapy versus ligation subgroup results (pooled MD = -1.29, 95% CI (-1.62, -0.97), p = 0.00001) (Figure 7).

Overall, these studies highlight the importance of considering the choice of the control group when evaluating the impact of sclerotherapy on QoL. While sclerotherapy can provide improvements in QoL, the specific control group used in the study can influence the perceived effectiveness of the treatment modality.

Then, we classified the inclusion studies from clinical severity subgroups based on the intervention mode or control type. The inclusion studies were divided into six subdivides: EVLA, placebo, deferred ablation, sapheno-femoral ligation, RFA, and stripping technique.

**Figure 8:**
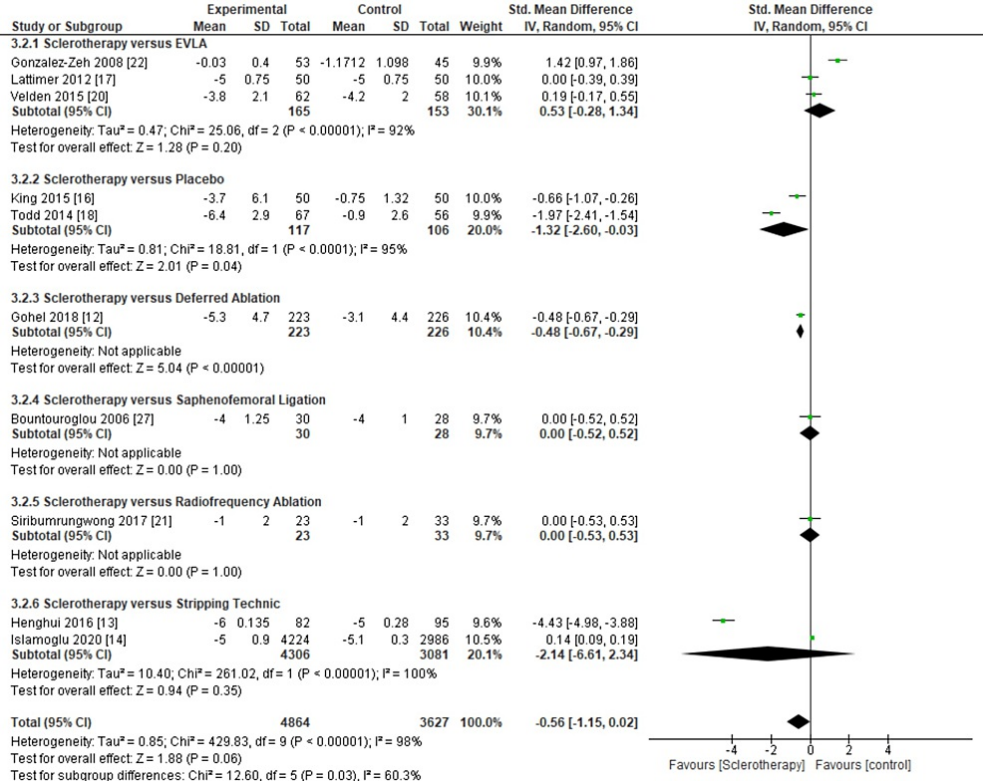
Meta-analysis based on the type of control for clinical severity outcomes. This figure was created by AAr, one of the authors of this article.

From the results of the meta-analysis, placebo and deferred ablation subgroup provides more significant results, for sclerotherapy versus placebo subgroup show significant results in sclerotherapy group (pooled MD = -1.32, 95% CI (-2.60, -0.03), p < 0.00001, I^2^ = 95%), and sclerotherapy versus deferred ablation subgroup show significant results in sclerotherapy (pooled MD = -0.48, 95% CI (-0.67, -0.29), p < 0.00001) compared to the EVLA, sapheno-femoral ligation, RFA, and stripping subgroup (Figure 8).

Next, we classified the inclusion studies from closure rate subgroups based on the mode of intervention or type of control. In this aspect, the inclusion studies were divided into eight subdivide, there are placebo, stripping, laser, mechanochemical, deferred ablation, EVLA, invagination, and sapheno-femoral ligation.

**Figure 9:**
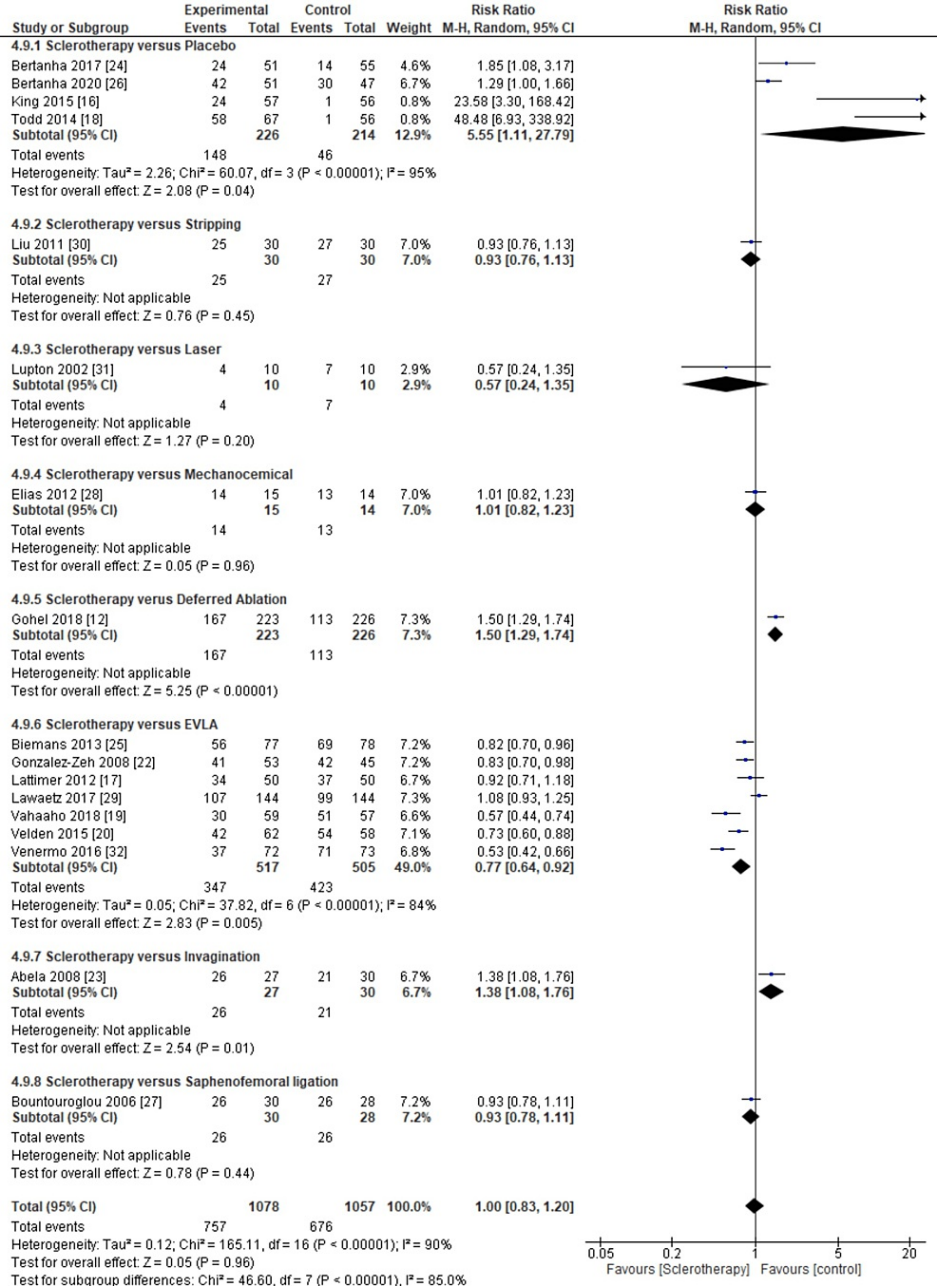
Meta-analysis based on the type of control for closure rate. This figure was created by FN, one of the authors of this article.

From the results of the meta-analysis, deferred ablation, EVLA, and invagination subgroup provide more significant effects. Meta-analysis results significant in the control group for sclerotherapy versus deferred subgroup (pooled RR = 1.50, 95% CI (1.29, 1.74), p < 0.00001) and sclerotherapy versus the EVLA subgroup show significant results in sclerotherapy group (pooled RR = 0.77, 95% CI (0.64, 0.9), p < 0.00001, I^2^ = 84%), and sclerotherapy versus invagination subgroup show significant results in control group (pooled RR = 1.38, 95% CI (1.08, 1.76), p = 0.01) (Figure 9).

Furthermore, we classified the inclusion studies from the closure rate subgroup based on the duration of outcome measures. The inclusion studies were divided into seven subdivisions: ≤ 1 month, 1.5 months, two months, three months, six months, 12 months, and 60 months.

**Figure 10:**
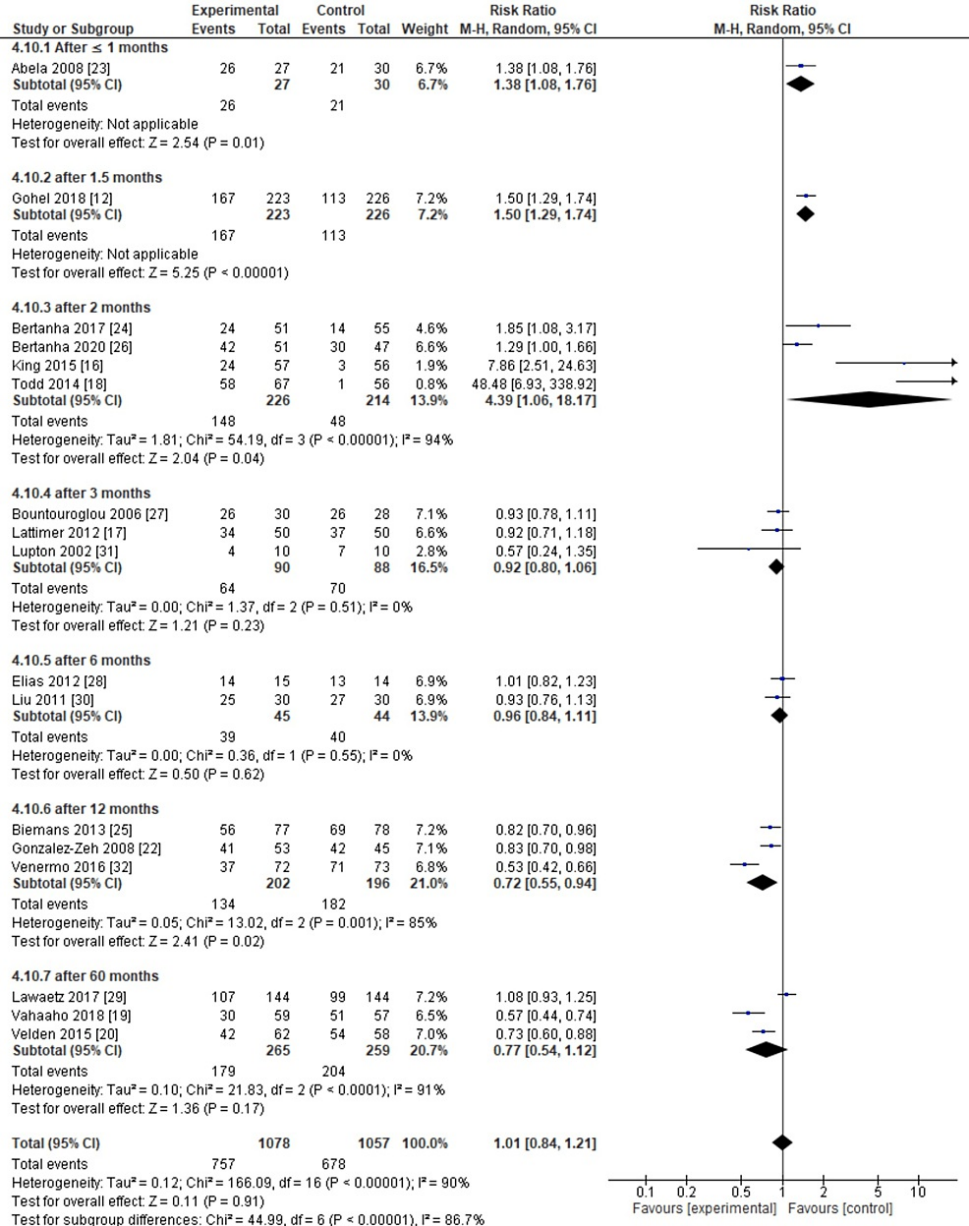
Meta-analysis based on the duration of outcome measures for closure rate. This figure was created by FN, one of the authors of this article.

From the meta-analysis results, the ≤1 month, 1.5 months, two months, and 12 months subgroup provides more significant effects. Meta-analysis results significantly in the control group for sclerotherapy after ≤1 months subgroup (pooled RR = 1.38, 95% CI (1.08, 1.76), p = 0.01) and sclerotherapy after 1.5-month subgroup show significant results in the control group (pooled RR = 1.50, 95% CI (1.29, 1.74), p < 0.00001), and then at this point sclerotherapy after two months subgroup show significant results in control group (pooled RR = 4.39, 95% CI (1.06, 18.17), p < 0.00001, I^2 ^= 94%). Additionally, sclerotherapy after 12-month subgroup show significant results in the intervention group (pooled RR = 0.72, 95% CI (0.55, 0.94), p = 0.001, I^2 ^= 91%) (Figure 10).

Discussion

Sclerotherapy improved the QoL for patients with varicose veins. A study by Rabe et al. found that sclerotherapy significantly improved symptoms such as pain, heaviness, and swelling, leading to an overall improvement in the QoL for patients. This improvement was sustained over 12 months [34].

Regarding clinical severity outcomes, sclerotherapy is effective in reducing the severity of varicose veins. A study by Rabe et al. demonstrated that sclerotherapy significantly reduced the clinical severity score, considering factors such as pain, swelling, and skin changes. This clinical severity reduction improved QoL and decreased QoL 12 months post-treatment [34].

Sclerotherapy has also been effective in achieving closure of treated veins. A study by Weiss et al. compared the closure rates of sclerotherapy with other treatment modalities, such as EVLA and foam sclerotherapy. The study found that sclerotherapy had comparable or even higher closure rates compared to EVLA and foam sclerotherapy. This suggests that sclerotherapy can be an effective treatment option for achieving vein closure [35].

Sclerotherapy works by causing fibrosis and closure of treated veins, improving QoL, and reducing clinical severity. It has also been effective in achieving the closure of treated veins, making it a viable treatment option for varicose and telangiectatic leg veins [36].

Sclerotherapy is a widely used treatment for varicose and telangiectatic leg veins, but there is ongoing debate regarding the optimal duration of outcome measures to assess its effectiveness. One study by Weiss et al. investigated the impact of different durations of post-sclerotherapy compression on clinical outcomes. The study found that longer durations of compression, specifically two weeks compared to one week, resulted in significantly better outcomes regarding vessel disappearance and reduction in pigmentation. This intervention suggests that the duration of outcome measures can influence the effectiveness of sclerotherapy, with longer durations of compression leading to improved outcomes [35].

Sclerotherapy is a commonly used treatment for varicose veins, and its impact on QoL can vary depending on the choice of control group. A study by Rasmussen et al. compared the effects of sclerotherapy with different control groups, including EVLA, placebo, deferred ablation, sapheno-femoral ligation, and stripping technique. The study found that sclerotherapy had similar improvements in QoL compared to EVLA, deferred ablation, sapheno-femoral ligation, and stripping technique. However, sclerotherapy had significantly better QoL outcomes compared to placebo. These findings suggest that the choice of a control group can influence the perceived effectiveness of sclerotherapy in improving QoL [37].

Sclerotherapy is a widely used treatment for varicose veins, but the choice of the control group can influence the clinical severity outcomes. A study by Rasmussen et al. compared the efficacy of sclerotherapy with different control groups, including EVLA, placebo, deferred ablation, sapheno-femoral ligation, RFA, and stripping technique. The study found that sclerotherapy had clinical severity outcomes similar to EVLA, deferred ablation, sapheno-femoral ligation, and RFA. However, sclerotherapy had significantly better clinical severity outcomes compared to placebo and stripping techniques. These findings suggest that the choice of a control group can impact the perceived effectiveness of sclerotherapy in terms of clinical severity improvement [37].

Another study by Kalteis et al. compared the effects of high ligation combined with stripping and EVLA on QoL. The study found that both procedures significantly improved QoL, with no significant differences between the two groups. This suggests that high ligation with stripping and EVLA can positively affect the QoL for patients with varicose veins [38].

Sclerotherapy provides a viable alternative to conventional surgery and EVLA. Other findings found in our study are as follows:

1) Sclerotherapy is the most effective therapy for reducing clinical severity in lower limb venous insufficiency patients.

2) Sclerotherapy is the most effective therapy for increasing QoL if we use VEINES-QOL and AVVS as tools for measuring QoL.

3) Sclerotherapy is a more effective therapy for increased QoL than placebo, deferred ablation, and ligation therapy.

4) Sclerotherapy is a more effective therapy for reducing clinical severity than placebo and deferred ablation therapy.

5) Sclerotherapy is a more effective therapy for increasing the closure than EVLA therapy.

6) Sclerotherapy is the most effective therapy for increasing the closure rate for 12-month outcome measures.

Despite the positive outcomes observed in this study, several areas require further investigation. Firstly, more studies are needed to determine the optimal duration of outcome measures for assessing the effectiveness of sclerotherapy in CVI. Standardizing the timing of follow-up assessments will allow for better comparison and interpretation of results. Additionally, the different types of sclerosing agents and their efficacy in treating CVI need to be evaluated. This will help identify the most effective agents and guide clinical decision-making. Furthermore, long-term follow-up studies are necessary to assess the durability of the treatment effects and the potential for recurrence. Moreover, exploring the cost-effectiveness of sclerotherapy compared to other treatment options is essential. Evaluating the economic impact of sclerotherapy will provide valuable information for healthcare providers and policymakers in making informed decisions regarding the allocation of resources.

Strength and Limitation

This review is the first study to discuss sclerotherapy and its correlation to QoL, clinical severity, and closure rate among patients with lower limb venous insufficiency. We conducted this study because the available studies still need to be focused on QoL, clinical severity, and closure rate among patients with lower limb venous insufficiency.

This study discusses the correlation between sclerotherapy effects on QoL, clinical severity, and closure rate in lower limb venous insufficiency patients. Due to variations in our journals (Table 2), such as sample size, the type of control, the duration of outcomes measures, and the scale used, we divided this meta-analysis into various subgroups.

The risk of bias in our inclusion studies mostly has some concerns in Domain 2 (Figure 2). Domain 2 is used to assess due to deviations from the intended interventions. Most of the journals need more information about deviations from the planned interventions. Therefore, we classified the journals with some concerns.

## Conclusions

Based on the systematic review and meta-analysis we have carried out, we can conclude that the analysis of randomized controlled trials and clinical trials revealed that sclerotherapy significantly improved patient satisfaction, clinical resolution, QoL, and closure rates compared to control interventions. However, high heterogeneity was observed in the meta-analysis results, indicating the need for further research to address this variability. The findings of this study contribute to the existing body of knowledge on the treatment options for CVI and highlight the potential of sclerotherapy as an effective treatment modality. Sclerotherapy offers a minimally invasive approach that can improve the appearance of veins and reduce symptoms associated with venous insufficiency. Sclerotherapy has shown promising results in treating CVI. However, further research is needed to optimize its effectiveness, standardize outcome measures, compare different sclerosing agents, assess long-term outcomes, and evaluate cost-effectiveness.

## Appendices

First appendix: study selection

**Table 3:** The Cochrane Central Register of Controlled Trials. This figure was created by AA, one of the authors of this article.

No	Search	Result
#1	Telangiectasia OR reticular vein OR spider vein OR varicose vein OR ulcer OR varicose vein trunk OR chronic venous insufficiency OR trophic ulcer	23755
#2	Sclerotherapy OR saline-sclerotherapy OR foam-sclerotherapy OR glycerin-sclerotherapy OR ultrasound-guided sclerotherapy	13144
#3	Placebo OR standard therapy	46872
#4	Patient satisfaction OR clinical resolution OR quality of life OR adverse event OR clinical improvement OR pain OR hyperpigmentation of skin	515736
#5	Lower limb OR great saphenous vein OR superficial OR small saphenous vein OR perforator vein	24595
#6	#1 AND #2 AND #3 AND #4 AND #5	32
Total: 32		

**Table 4:** PubMed. This figure was created by AA, one of the authors of this article.

No	Search	Result
#1	Telangiectasia OR reticular vein OR spider vein OR varicose vein(MeSH Terms)	45972
#2	Sclerotherapy OR saline-sclerotherapy OR foam-sclerotherapy OR glycerin-sclerotherapy OR ultrasound-guided sclerotherapy(MeSH Terms)	9823
#3	Patient satisfaction OR clinical resolution OR quality of life OR adverse event OR clinical improvement OR pain OR hyperpigmentation of skin	3314427
#4	Lower limb OR great saphenous vein OR superficial OR small saphenous vein OR perforator vein	374436
#5	(((Telangiectasia OR reticular vein OR ulcer OR varicose vein OR spider vein(MeSH Terms)) AND (Sclerotherapy OR saline-sclerotherapy OR foam-sclerotherapy OR glycerin-Sclerotherapy OR ultrasound-Guided Sclerotherapy(MeSH Terms))) AND (patient satisfaction OR clinical resolution OR quality of life OR adverse event OR side effect OR clinical improvement OR pain OR hyperpigmentation of skin)) AND (lower limb OR great saphenous vein OR superficial OR small saphenous vein OR perforator vein)	379
#6	(((Telangiectasia OR reticular vein OR ulcer OR varicose vein OR spider vein(MeSH Terms)) AND (Sclerotherapy OR saline-sclerotherapy OR foam-sclerotherapy OR glycerin-Sclerotherapy OR ultrasound-Guided Sclerotherapy(MeSH Terms))) AND (patient satisfaction OR clinical resolution OR quality of life OR adverse event OR side effect OR clinical improvement OR pain OR hyperpigmentation of skin)) AND (lower limb OR great saphenous vein OR superficial OR small saphenous vein OR perforator vein) AND ((ffrft(Filter)) AND (clinicaltrial(Filter) OR randomizedcontrolledtrial(Filter))) Filters: Free full text, Clinical Trial, Randomized Controlled Trial	29
Total: 29		

Google Scholar

Sclerotherapy Varicose OR vein OR reticular OR vein OR spider OR vein OR telangiectasia "trial" -Review

Total: 2070

## References

[1] Al Shammeri O, AlHamdan N, Al-Hothaly B, Midhet F, Hussain M, Al-Mohaimeed A (2014). Chronic venous insufficiency: prevalence and effect of compression stockings. Int J Health Sci (Qassim).

[2] Santiago F (2023). Quality of life in chronic venous disease: bridging the gap between patients and physicians. Clin Drug Investig.

[3] Ortega MA, Fraile-Martínez O, García-Montero C (2021). Understanding chronic venous disease: a critical overview of its pathophysiology and medical management. J Clin Med.

[4] Mansilha A, Sousa J (2018). Pathophysiological mechanisms of chronic venous disease and implications for venoactive drug therapy. Int J Mol Sci.

[5] Orhurhu V, Chu R, Xie K (2021). Management of lower extremity pain from chronic venous insufficiency: a comprehensive review. Cardiol Ther.

[6] Dialsingh I, Mohammed SR, Medford RS, Budhoo E, White-Gittens I, Maharaj D (2022). Conservative therapy significantly reduces patients' chronic venous disease symptoms: a Caribbean insight into the VEIN Act Program. Phlebology.

[7] Worthington-Kirsch RL (2005). Injection sclerotherapy. Semin Intervent Radiol.

[8] Rabe E, Breu FX, Flessenkämper I (2021). Sclerotherapy in the treatment of varicose veins. Der Hautarzt.

[9] Bergan JJ, Pascarella L (2005). Severe chronic venous insufficiency: primary treatment with sclerofoam. Semin Vasc Surg.

[10] Ho VT, Adkar SS, Harris EJ Jr (2022). Systematic review and meta-analysis of management of incompetent perforators in patients with chronic venous insufficiency. J Vasc Surg Venous Lymphat Disord.

[11] Shivhare P, Haidry N, Sah N (2022). Comparative evaluation of efficacy and safety of the diode laser (980 nm) and sclerotherapy in the treatment of oral vascular malformations. Int J Vasc Med.

[12] Gohel MS, Heatley F, Liu X (2018). A randomized trial of early endovenous ablation in venous ulceration. N Engl J Med.

[13] Yin H, He H, Wang M (2017). Prospective randomized study of ultrasound-guided foam sclerotherapy combined with great saphenous vein high ligation in the treatment of severe lower extremity varicosis. Ann Vasc Surg.

[14] Islamoglu F (2020). Varicose vein surgery versus foam sclereotherapy to prevent extension of venous reflux with time. Ann Circ.

[15] Kalodiki E, Azzam M, Schnatterbeck P, Geroulakos G, Lattimer CR (2019). The discord outcome analysis (DOA) as a reporting standard at three months and five years in randomised varicose vein treatment trials. Eur J Vasc Endovasc Surg.

[16] King JT, O'Byrne M, Vasquez M, Wright D (2015). Treatment of truncal incompetence and varicose veins with a single administration of a new polidocanol endovenous microfoam preparation improves symptoms and appearance. Eur J Vasc Endovasc Surg.

[17] Lattimer CR, Azzam M, Kalodiki E, Shawish E, Trueman P, Geroulakos G (2012). Cost and effectiveness of laser with phlebectomies compared with foam sclerotherapy in superficial venous insufficiency. Early results of a randomised controlled trial. Eur J Vasc Endovasc Surg.

[18] Todd KL, Wright DI (2014). The VANISH-2 study: a randomized, blinded, multicenter study to evaluate the efficacy and safety of polidocanol endovenous microfoam 0.5% and 1.0% compared with placebo for the treatment of saphenofemoral junction incompetence. Phlebology.

[19] Vähäaho S, Halmesmäki K, Albäck A, Saarinen E, Venermo M (2018). Five-year follow-up of a randomized clinical trial comparing open surgery, foam sclerotherapy and endovenous laser ablation for great saphenous varicose veins. Br J Surg.

[20] van der Velden SK, Biemans AA, De Maeseneer MG (2015). Five-year results of a randomized clinical trial of conventional surgery, endovenous laser ablation and ultrasound-guided foam sclerotherapy in patients with great saphenous varicose veins. Br J Surg.

[21] Siribumrungwong B, Noorit P, Wilasrusmee C, Teerawattananon Y, Thakkinstian A (2017). Quality of life after great saphenous vein ablation in Thai patients with great saphenous vein reflux. Asian J Surg.

[22] Gonzalez-Zeh R, Armisen R, Barahona S (2008). Endovenous laser and echo-guided foam ablation in great saphenous vein reflux: one-year follow-up results. J Vasc Surg.

[23] Abela R, Liamis A, Prionidis I, Mathai J, Gorton L, Browne T, Panayiotopoulos Y (2008). Reverse foam sclerotherapy of the great saphenous vein with sapheno-femoral ligation compared to standard and invagination stripping: a prospective clinical series. Eur J Vasc Endovasc Surg.

[24] Bertanha M, Jaldin RG, Moura R (2017). Sclerotherapy for reticular veins in the lower limbs a triple-blind randomized clinical trial. JAMA Dermatol.

[25] Biemans AA, Kockaert M, Akkersdijk GP (2013). Comparing endovenous laser ablation, foam sclerotherapy, and conventional surgery for great saphenous varicose veins. J Vasc Surg.

[26] Bertanha M, Yoshida WB, Bueno de Camargo PA, Moura R, Reis de Paula D, Padovani CR, Sobreira ML (2021). Polidocanol plus glucose versus glucose alone for the treatment of telangiectasias: triple blind, randomised controlled trial (PG3T). Eur J Vasc Endovasc Surg.

[27] Bountouroglou DG, Azzam M, Kakkos SK, Pathmarajah M, Young P, Geroulakos G (2006). Ultrasound-guided foam sclerotherapy combined with sapheno-femoral ligation compared to surgical treatment of varicose veins: early results of a randomised controlled trial. Eur J Vasc Endovasc Surg.

[28] Elias S, Raines JK (2012). Mechanochemical tumescentless endovenous ablation: final results of the initial clinical trial. Phlebology.

[29] Lawaetz M, Serup J, Lawaetz B, Bjoern L, Blemings A, Eklof B, Rasmussen L (2017). Comparison of endovenous ablation techniques, foam sclerotherapy and surgical stripping for great saphenous varicose veins. Extended 5-year follow-up of a RCT. Int Angiol.

[30] Liu X, Jia X, Guo W (2011). Ultrasound-guided foam sclerotherapy of the great saphenous vein with sapheno-femoral ligation compared to standard stripping: a prospective clinical study. Int Angiol.

[31] Lupton JR, Alster TS, Romero P (2002). Clinical comparison of sclerotherapy versus long-pulsed Nd:YAG laser treatment for lower extremity telangiectases. Dermatol Surg.

[32] Venermo M, Saarinen J, Eskelinen E (2016). Randomized clinical trial comparing surgery, endovenous laser ablation and ultrasound-guided foam sclerotherapy for the treatment of great saphenous varicose veins. Br J Surg.

[33] Goldman MP (2002). Treatment of varicose and telangiectatic leg veins: double-blind prospective comparative trial between aethoxyskerol and sotradecol. Dermatol Surg.

[34] Rabe E, Breu FX, Cavezzi A (2014). European guidelines for sclerotherapy in chronic venous disorders. Phlebology.

[35] Weiss RA, Sadick NS, Goldman MP, Weiss MA (1999). Post-sclerotherapy compression: controlled comparative study of duration of compression and its effects on clinical outcome. Dermatol Surg.

[36] Gloviczki P, Comerota AJ, Dalsing MC (2011). The care of patients with varicose veins and associated chronic venous diseases: clinical practice guidelines of the Society for Vascular Surgery and the American Venous Forum. J Vasc Surg.

[37] Rasmussen LH, Lawaetz M, Bjoern L, Vennits B, Blemings A, Eklof B (2011). Randomized clinical trial comparing endovenous laser ablation, radiofrequency ablation, foam sclerotherapy and surgical stripping for great saphenous varicose veins. Br J Surg.

[38] Kalteis M, Berger I, Messie-Werndl S, Pistrich R, Schimetta W, Pölz W, Hieller F (2008). High ligation combined with stripping and endovenous laser ablation of the great saphenous vein: early results of a randomized controlled study. J Vasc Surg.

